# Prevention of Type 2 Diabetes by lifestyle intervention in an Australian primary health care setting: Greater Green Triangle (GGT) Diabetes Prevention Project

**DOI:** 10.1186/1471-2458-7-249

**Published:** 2007-09-19

**Authors:** Tiina Laatikainen, James A Dunbar, Anna Chapman, Annamari Kilkkinen, Erkki Vartiainen, Sami Heistaro, Ben Philpot, Pilvikki Absetz, Stephen Bunker, Adrienne O'Neil, Prasuna Reddy, James D Best, Edward D Janus

**Affiliations:** 1 Greater Green Triangle University Department of Rural Health, Flinders and Deakin Universities, PO Box 423, Warrnambool, Victoria, 3280, Australia; 2 National Public Health Institute, Mannerheimintie 166, FI – 00300, Helsinki, Finland; 3 Department of Medicine, University of Melbourne, St Vincent's Hospital, PO Box 2900, Fitzroy, Victoria, 3065, Australia; 4 Department of Medicine, University of Melbourne, Western Hospital, Footscray, Victoria, 3011 Australia

## Abstract

**Background:**

Randomised controlled trials demonstrate a 60% reduction in type 2 diabetes incidence through lifestyle modification programmes. The aim of this study is to determine whether such programmes are feasible in primary health care.

**Methods:**

An intervention study including 237 individuals 40–75 years of age with moderate or high risk of developing type 2 diabetes. A structured group programme with six 90 minute sessions delivered during an eight month period by trained nurses in Australian primary health care in 2004–2006. Main outcome measures taken at baseline, three, and 12 months included weight, height, waist circumference, fasting plasma glucose and lipids, plasma glucose two hours after oral glucose challenge, blood pressure, measures of psychological distress and general health outcomes. To test differences between baseline and follow-up, paired t-tests and Wilcoxon rank sum tests were performed.

**Results:**

At twelve months participants' mean weight reduced by 2.52 kg (95% confidence interval 1.85 to 3.19) and waist circumference by 4.17 cm (3.48 to 4.87). Mean fasting glucose reduced by 0.14 mmol/l (0.07 to 0.20), plasma glucose two hours after oral glucose challenge by 0.58 mmol/l (0.36 to 0.79), total cholesterol by 0.29 mmol/l (0.18 to 0.40), low density lipoprotein cholesterol by 0.25 mmol/l (0.16 to 0.34), triglycerides by 0.15 mmol/l (0.05 to 0.24) and diastolic blood pressure by 2.14 mmHg (0.94 to 3.33). Significant improvements were also found in most psychological measures.

**Conclusion:**

This study provides evidence that a type 2 diabetes prevention programme using lifestyle intervention is feasible in primary health care settings, with reductions in risk factors approaching those observed in clinical trials.

**Trial Number:**

Current Controlled Trials ISRCTN38031372

## Background

It is widely recognised that the incidence of type 2 diabetes is high and increasing both in Australia [1] and throughout the world [2]. Type 2 diabetes is a chronic and costly disease associated with premature mortality and high rates of health service utilisation linked with its complications: cardiovascular disease, retinopathy, renal failure and neuropathy [3].

The risk factors associated with type 2 diabetes onset are to a large degree preventable. Lifestyle modification, particularly weight loss and physical activity, can significantly reduce the risk of type 2 diabetes [4-6]. Diabetes prevention trials using lifestyle modification have been proven effective in reducing the risk of developing type 2 diabetes [4-6], and have shown to be even more effective than pharmacological interventions [6-9]. The Finnish Diabetes Prevention Study [4] investigated the role of lifestyle interventions in the progression of type 2 diabetes among individuals with impaired glucose tolerance. Results demonstrated that the incidence of type 2 diabetes decreased by 58% in the intervention group compared with the control group, a finding which was directly associated with lifestyle modification. Two other studies have demonstrated similar results [5,6]. Randomised controlled trials with one-to-one counselling and trials using drugs are expensive. The lifestyle interventions have all lasted several years [6,10] and for example in the DPS, the median number of counselling sessions during a 3-year intervention was 20 [4]. The cost per patient per year in the DREAM trial [7] where rosiglitazone and ramipril treatments were used was approximately USD 4700. To determine whether the results obtained in clinical trials could be replicated in "real world" primary health care settings with limited resources and existing personnel, the GOAL intervention study [11], a lifestyle implementation trial using a structured group programme, was designed in Finland. Whilst the implementation of lifestyle modification interventions in routine health care pose a great challenge [12], the results of this programme have demonstrated that group lifestyle counselling can be effective and feasible in 'real world' settings for individuals with an elevated risk of type 2 diabetes [13].

Given that the efficacy of lifestyle modification treatments have been well established by earlier diabetes prevention trials [4,6], the need for an additional randomised controlled trial study design in the current programme was unnecessary. Further, it was considered unethical not to offer this effective treatment to all individuals in the programme. More appropriately, as the evidence supporting the intervention is so strong, the purpose was to examine the implementation of the intervention into routine clinical practice [13,14].

The aim of this study was to evaluate the feasibility of the structured group programme for lifestyle modification in Australian primary care settings. The results of the first three months of intervention have been published earlier [15].

## Methods

### Design

This study was developed and evaluated as an implementation trial in a practical setting in order to establish whether it is possible to achieve findings comparable to the Diabetes Prevention Study [4]. A longitudinal pre test and post test study design was used for examining changes in clinical outcome measures [13].

### Recruitment

This study was carried out in the Greater Green Triangle of Southwest Victoria and Southeast South Australia in 2004–2006 using General Practices in Hamilton, Horsham and Mount Gambier.

Participants were patients presenting at local General Practices and were screened opportunistically by study nurses in reception and waiting areas. The Diabetes Risk Score tool [16], developed from population based epidemiological studies, was used to identify patients at high risk of developing type 2 diabetes. A score of 12 or more was used as a recruitment criterion. More than 1,500 people were approached, and of those approximately two thirds were willing to be screened. Individuals with cancer, recent myocardial infarction or stroke, cognitive impairment, substance abuse, pregnancy or a previous type 2 diabetes diagnoses were excluded from the study. Of the 523 subjects whose score was ≥12, 343 subjects (65.6%) were willing to participate in the study.

Those (n = 32) who were diagnosed with type 2 diabetes based on their baseline fasting plasma glucose value (≥7.0 mmol/l) and/or two-hour oral glucose tolerance test (>11.0 mmol/l) were excluded from the analyses [17]. In total, 311 participants (88 males and 223 females) aged 40–75 years were eligible to participate. The mean risk score for eligible participants was 15.7 equating to a one in three chance of developing type 2 diabetes during the following 10 years [16].

### Intervention

The intervention model used in the study was based on the diabetes prevention project in the Finnish GOAL study described in detail elsewhere [11]. Briefly, the intervention consists of six structured 90 minute group sessions over eight months using the Health Action Process Approach.

The first five sessions occurred within the first three months, with two week intervals between sessions. The last session took place at eight months. The sessions were facilitated by specially trained study nurses, dietitians and physiotherapists. A goal setting approach was used to motivate individuals to progress from intention to actual behaviour change. Regular self-assessment was used to empower participants to take responsibility for their own decisions and to make informed choices. Session content for diet and physical activity was based on Dietary Guidelines for Australian Adults [18] and National Physical Activity Guidelines for Adults [19]. Social support was enhanced by the group setting and by encouraging participants to seek support from their own social networks. Intervention targets followed the lifestyle targets in the Finnish Diabetes Prevention Study [4] aiming to reduce weight, total and saturated fat intake and increase fibre intake and physical activity.

### Measurements

Clinical measurements included height, weight, waist, hip and blood pressure measurements, all of which were taken by study nurses before the intervention, at three months, and at one year. Height, weight, waist, hip and blood pressure were measured following the European Health Risk Monitoring protocol [20]. Blood pressure was measured using a mercury sphygmomanometer.

Fasting venous samples were drawn at the baseline, at three months, and at one year. All participants were required to fast overnight prior to each clinical test for a minimum of eight hours. A quality control was performed before each clinical test by asking participants the duration of their fasting period. Fasting hours ranged between 9–17 hours. Oral glucose tolerance tests using 75 g glucose was performed at baseline and twelve months. The ranges used for impaired glucose values were fasting glucose 6.1 to 6.9 mmol/l (impaired fasting glucose, IFG) or plasma glucose concentration 7.8 to 11.0 mmol/l two hours after the oral administration of 75 g of glucose (impaired glucose tolerance, IGT) [17,20]. Frozen plasma samples were transferred to Flinders Medical Centre clinical trials laboratory. A Hitachi 917 clinical chemical analyser (Roche Diagnostic, Australia) was used to measure plasma glucose, total cholesterol, triglycerides and high density lipoprotein (HDL) cholesterol by standard enzymatic methods. A direct HDL-cholesterol method was used. Low density lipoprotein (LDL) cholesterol was calculated using the Friedewald equation unless triglycerides were greater than 4.5 mmol/l. The Flinders Medical Centre Clinical Trials laboratory participates in Centers for Disease Control and Prevention Lipid Standardisation Program.

At baseline and 12 months, participants were assessed using the Kessler 10 Psychological Distress Scale (K-10) [21] and Hospital Anxiety and Depression Scale (HADS) [22]. General health was assessed using Short Form 36 (SF-36v2) [23].

### Statistical analyses

Sample size calculations to detect a 5% reduction in main outcome variables such as weight, serum cholesterol, blood glucose etc. with 80% power at the 5% significance level were carried out.

Statistical analyses were performed using SPSS Version 14. To test differences between baseline and follow-up, paired t-tests were used. For psychosocial data, Wilcoxon rank sum tests were performed. Effect sizes were calculated for changes in psychological and general health measures by dividing the change in means by the standard deviation of the baseline mean [24].

### Ethics

Participants gave written consent to participate. The study was approved by the Flinders University Clinical Research Ethics Committee (reference number 105/034).

## Results

### Baseline characteristics of completers and non-completers

Out of 311 who started in the intervention, 237 (65 males and 172 females) attended both the baseline and 12 month clinical tests and at least one group session. This was the definition of completion. Forty-three percent of completers participated in all six sessions and only 9.7% in three or less sessions. Reasons for non-completion were: lack of transport, fuel costs, time constraints, poor literacy and health conditions.

The baseline characteristics of those who completed the program were compared with those who did not. Compared with completers, non-completers had significantly higher waist circumferences, lower levels of education and higher scores on measures of psychological distress, anxiety and depression (Table 1). In most domains of the SF-36v2, non-completers reported poorer health.

**Table 1:** Baseline characteristics of completers and non-completers

Characteristic	Completers (n = 237) mean (SD)	Non-completers (n = 74) mean (SD)	P value
Age (yr)	56.7 (8.7)	57.2 (9.5)	0.705
Weight (kg)	91.7 (17.7)	95.3 (19.6)	0.145
Body-mass index (kg/m^2^)	33.5 (5.9)	34.7 (6.9)	0.144
Waist circumference (cm)	104.9 (13.0)	109.6 (14.9)	0.009
Hip circumference (cm)	115.7 (12.5)	117.6 (13.9)	0.257
Plasma glucose (mmol/l)			
Fasting	5.53 (0.54)	5.46 (0.51)	0.298
2 Hr oral glucose challenge	6.71 (1.68)	6.61 (1.72)	0.644
Serum lipids (mmol/l)			
Total cholesterol	5.67 (1.15)	5.42 (0.98)	0.102
Low-density lipoprotein cholesterol	3.44 (0.99)	3.25 (0.80)	0.097
High-density lipoprotein cholesterol	1.34 (0.37)	1.26 (0.34)	0.082
Triglycerides	1.94 (1.05)	2.02 (1.14)	0.569
Blood pressure (mm Hg)			
Systolic	132.1 (17.1)	135.0 (18.8)	0.216
Diastolic	81.0 (9.9)	82.6 (11.7)	0.265
HADS anxiety	3.6 (3.3)	5.4 (4.0)	<0.0005
HADS depression	2.8 (2.9)	4.1 (3.9)	0.014
Kessler 10 (psychological distress)	15.3 (5.4)	19.0 (8.0)	<0.0005
SF-36 v2			
Physical functioning	78.3 (18.6)	66.3 (26.3)	0.001
Role limitation physical	78.6 (22.4)	67.3 (29.6)	0.008
Bodily pain	63.0 (23.9)	57.8 (27.5)	0.107
General health perception	65.9 (18.6)	61.0 (18.9)	0.034
Vitality	56.4 (19.2)	50.1 (23.8)	0.023
Social functioning	83.2 (20.3)	75.2 (27.3)	0.055
Role limitation emotional	85.4 (21.0)	76.9 (27.3)	0.026
Mental health	75.7 (17.0)	66.5 (20.5)	<0.0005
			
Years of education	12.1 (3.4)	10.7 (3.1)	0.002

### Clinical outcomes for completers

Between baseline and twelve months, statistically significant improvements were observed in participants' mean clinical indicators except systolic blood pressure (Table 2). The greatest improvement (8.6%) was seen in plasma glucose after two hours oral glucose challenge. Total cholesterol declined 5.1%, LDL cholesterol 7.3% and triglycerides 7.6%, whilst HDL cholesterol increased 4.4%. Waist, weight and diastolic blood pressure declined 4.0%, 2.7% and 2.6%, respectively. Seventy-five percent of participants experienced some waist reduction and 68% experienced weight reduction.

**Table 2:** Changes in clinical outcome in baseline, three months and 12 months

	n	Baseline mean (SD)	Change from baseline to 3 months* mean (95% CI)	Change from baseline to 12 months mean (95% CI)	Change at 12 months %
Weight (kg)	237	91.7 (17.7)	-2.38 (-2.79,-1.98)	-2.52 (-3.19,-1.85)	-2.7
Body-mass index (kg/m^2^)	237	33.5 (5.9)	-0.88 (-1.03,-0.74)	-0.93 (-1.17,-0.69)	-2.8
Waist circumference (cm)	236	104.9 (13.0)	-3.16 (-3.72,-2.60)	-4.17 (-4.87,-3.48)	-4.0
Hip circumference (cm)	236	115.7 (12.5)	-1.56 (-2.04,-1.07)	-2.65 (-3.26,-2.05)	-2.3
Plasma glucose (mmol/l)					
Fasting	237	5.53 (0.54)	0.00 (-0.07,0.08)	-0.14 (-0.20,-0.07)	-2.5
2 Hr oral glucose challenge	232	6.70 (1.69)	-	-0.58 (-0.79,-0.36)	-8.6
Serum lipids (mmol/l)					
Total cholesterol	237	5.67 (1.15)	-0.21 (-0.32,-0.09)	-0.29 (-0.40,-0.18)	-5.1
Low-density lipoprotein cholesterol	229	3.44 (0.99)	-0.16 (-0.25,-0.07)	-0.25 (-0.34,-0.16)	-7.3
High-density lipoprotein cholesterol	237	1.34 (0.37)	0.01 (-0.02,0.03)	0.06 (0.03,0.09)	+4.4
Triglycerides	237	1.90 (1.00)	-0.10 (-0.20,0.01)	-0.15 (-0.24,-0.05)	-7.6
Blood pressure (mmHg)					
Systolic	236	132.1 (17.1)	-1.67 (-3.3,-0.04)	-1.01 (-2.60,0.58)	-0.8
Diastolic	236	81.0 (9.9)	-0.99 (-2.18,0.20)	-2.14 (-3.33,-0.94)	-2.6

* At the 3 month clinical test, n value varied between 216 and 221.

Statistically significant improvements to weight, waist, total and LDL-cholesterol were evident after the first three months of the intervention and these changes were sustained at 12 months. Although not observed at 3 months, statistically significant improvements were found in fasting glucose, HDL-cholesterol, triglycerides and diastolic blood pressure at 12 months.

At baseline, 65.9% of participants had normal glucose values and 34.1% had impaired glucose values (IFG 9.5%, IGT 24.6%). At the 12 month clinical test, 78.0% had normal glucose values and 19.8% had impaired values (IFG 6.5%, IGT 13.4%). Out of 79 participants who had impaired values at baseline, five (2.2%) developed type 2 diabetes during the intervention and 42 (18.1%) reverted to normoglycemia.

### Psychological and general health outcomes

Between baseline and 12 month clinical tests, improvements in SF-36v2 domains of vitality and general health were moderate (Table 3). Small but statistically significant improvements were observed in the domains of bodily pain, physical functioning and mental health as well as for measures of psychological distress (Kessler 10, HADS).

**Table 3:** Mean scores and standard deviations of participant responses to psychological measures and proportion of participants whose scores improved between baseline and 12 month intervention

		n	Baseline mean (SD)	Change from baseline to 12 months mean (95% CI)	Effect size	P value
Kessler 10	Psychological distress	226	15.1 (5.2)	14.6 (5.6)	+0.10	0.002
HADS	Depression	224	2.8 (2.9)	2.5 (3.0)	+0.10	0.008
SF-36 v2	Physical Functioning	229	78.5 (18.1)	80.1 (19.9)	-0.09	0.012
	Role-Physical	230	78.7 (22.3)	78.3 (24.0)	+0.02	0.664
	Bodily Pain	232	63.0 (23.9)	66.4 (24.7)	-0.14	0.019
	General Health	229	65.9 (18.6)	71.3 (17.7)	-0.29	<0.0005
	Vitality	230	56.5 (19.2)	61.2 (18.9)	-0.25	<0.0005
	Social Functioning	232	83.1 (20.4)	82.7 (23.1)	+0.02	0.821
	Role-Emotional	230	85.3 (21.2)	85.7 (21.4)	-0.02	0.726
	Mental Health	230	75.9 (16.8)	78.4 (16.1)	-0.15	0.025

## Discussion

This study provides convincing evidence that a type 2 diabetes prevention programme using lifestyle intervention is feasible in Australian primary health care with reductions in risk factors approaching those observed in randomised controlled trials [4-6]. Results from the present study confirm that significant changes can be obtained in weight, waist, glucose and lipids, and psychological measures.

The implementation of this trial in a "real world" setting has allowed findings to be more generalisable to primary health care, as they have been performed in a setting in which future implementation is likely to occur [14]. Using the DPS sample as a reference population to predict a reduction in type 2 diabetes risk, by using a single pre-test and post-test study design [13], it can be estimated that the 4.0% decline in waist circumference in our study reduces the risk of type 2 diabetes by 40%. Alternatively, calculating the risk reduction using weight change the diabetes risk could reduce by 23%. Based on cohort studies, waist circumference is a better predictor for type 2 diabetes [25], making the risk prediction based on waist probably a more accurate estimate. Additionally, in this study the components of metabolic syndrome and the risk factors for cardiovascular disease were improved.

Compared with drug treatment that is largely long term, successful lifestyle changes have an impact beyond the intervention period [8,10]. The latest follow up of the Finnish Diabetes Prevention Study [10] shows that the impact of lifestyle modification will last at least several years after intervention and the reduction in diabetes incidence was still 36% lower in the intervention group compared with the control group after a median three years. There is no evidence about the long term impact of drug treatment and it could be assumed that when drug treatment is ended the treatment effect may disappear [8].

This is the first diabetes prevention study where the clinical significance of psychosocial risk factors were identified. When investigating the characteristics of completers and non-completers, it was found that at baseline non-completers had fewer years of education and greater levels of psychological distress. Future programmes, should give more attention to these factors when considering strategies to improve retention rates. In both the present and previous diabetes prevention studies, more women than men have attended the programmes [4-6,13]. More emphasis should therefore be put on identifying the barriers to male attendance. Although this intervention was feasible and effective for 60% of those who were screened positively for the risk of type 2 diabetes, there is a need to develop other approaches for those who may not take part in group interactive programmes.

Findings from earlier studies suggest that participant weight loss can be predicted by facilitator motivation and self-fulfilment [26]. It was also our experience that appropriate facilitator training and support are imperative.

## Conclusion

This study provides evidence that a type 2 diabetes prevention programme using lifestyle intervention is feasible in primary health care settings, with reductions in risk factors approaching those observed in clinical trials.

## Competing interests

The author(s) declare that they have no competing interests.

## Authors' contributions

TL initiated, organised, and supervised the study and contributed to the data analyses and writing the manuscript. JAD was the lead investigator and is the guarantor. AC coordinated the study and carried out data-acquisition. AK was involved in data analysis, interpretation and writing the manuscript. BP did statistical analysis. EV was involved in preparing the study design and writing the manuscript. SH was involved in project supervision and data-acquisition. PA assisted in the development of the study design and content of the intervention. SB and PR assisted in planning and collection of psychosocial data and helped in drafting the manuscript and interpreting the results. AO was involved in drafting the manuscript. JDB participated in planning the study and in the writing. EDJ supervised the study. All authors read and approved the final manuscript.

## Pre-publication history

The pre-publication history for this paper can be accessed here:


